# Polymorphism (-499C/G) in *DDAH2* promoter may act as a protective factor for metabolic syndrome: A case-control study in Azar-Cohort population

**DOI:** 10.20945/2359-3997000000391

**Published:** 2021-07-16

**Authors:** Elnaz Faramarzi, Younes Aftabi, Khalil Ansarin, Mohammad Hossein Somi, Neda Gilani, Ensiyeh Seyedrezazadeh

**Affiliations:** 1 Tabriz University of Medical Sciences Liver and Gastrointestinal Diseases Research Center Tabriz Iran Liver and Gastrointestinal Diseases Research Center, Tabriz University of Medical Sciences, Tabriz, Iran; 2 Tabriz University of Medical Sciences Tuberculosis and Lung Diseases Research Center Tabriz Iran Tuberculosis and Lung Diseases Research Center, Tabriz University of Medical Sciences, Tabriz, Iran; 3 Tabriz University of Medical Sciences Faculty of Health Department of Statistics and Epidemiology Tabriz Iran Department of Statistics and Epidemiology, Faculty of Health, Tabriz University of Medical Sciences, Tabriz, Iran

**Keywords:** *DDAH2*, rs805305, polymorphism, metabolic syndrome, cohort study

## Abstract

**Objective:**

Globally developing metabolic syndrome (MetS) prevalence as a major health problem can be related to multiple factors of genetic and environmental. Dimethylaminohydrolase 2 (DDAH2) is the main enzyme implicated in the cardiovascular system, which regulates the nitric oxide pathway. This study investigated the association of DDAH2 polymorphism −499C/G (rs805305) with the risk of MetS among the Azar-Cohort population.

**Subjects and methods:**

The occurrence of SNP rs805305 in the DDAH2 gene was tested using the PCR-RFLP method in 332 MetS cases and 294 healthy controls. Afterward, the association of the allele and genotypes with the risk of MetS and its components were examined.

**Results:**

The G allele and GC genotype were significantly associated with a reduced risk of MetS (P ≤ 0.001). Also, the dominant genetic model (GG+GC) significantly decreased the risk of MetS (P = 0.001), however, in sex subtypes MetS risk was significantly reduced in males before and in females after adjustment for age (P ≤ 0.02).

**Conclusion:**

The −499C/G polymorphism of DDAH2 may play a protective role and reduce MetS risk among the Azar-Cohort population.

## INTRODUCTION

Metabolic syndrome (MetS) is a bunch of various metabolic disorders, comprise obesity, insulin resistance, glucose metabolism faulty, and hypertension ( 1 ). Increasing risk of some chronic diseases including cardiovascular diseases, cancer, and diabetic mellitus attributed to metabolic syndrome as a new silent killer ( 2 ). MetS have become a noteworthy general medical issue worldwide and its prevalence is also dependent on defined criteria and a variety of regions. For instance, it is estimated its prevalence is 44.1% in Jordan, 28.9% in Turkey, and 30.4% in Iran ( 3 – 5 ). Despite the global prevalence increment of MetS, its etiology is not entirely recognized. Multiple risk factors, including obesity, insulin resistance, low-grade inflammation, oxidative stress, environmental, and genetic factors, are attributed to play major roles in MetS development ( 6 , 7 ).

Nowadays, the roles of genetic factors are more considered. In this sense, the reduction level of nitric oxide (NO) as a key bioactive molecule produced by nitric oxide synthase is critically involved in pathophysiological events of MetS. Dysregulation of the NO pathway is mainly attributed to increased levels of asymmetric dimethylarginine (ADMA). ADMA is originated from the proteolysis of various proteins with methylated arginine residues. It is an endogenous inhibitor of nitric oxide synthase (NOS) which is complicated in MetS pathogenesis ( 8 ). Moreover, ADMA degrades via the action of dimethylarginine dimethylaminohydrolase (DDAH) enzymes, that two isoforms of them have been determined: DDAH1 is believed to be associated with neuronal NOS (nNOS) and DDAH2 is thought to be related with tissue endothelial NOS (eNOS) expression ( 9 ). These enzymes are encoded on chromosomes 1p22 and 6p21.3 ( 10 ). As reported, with high oxidative stress conditions in MetS, the activity of *DDAH2* reduced ( 11 ). More than 70% of the ADMA level is metabolized by DDAH 2, hence reduction of DDAH2 activity induces ADMA level elevation, which in turn decreases NO signaling and according to that increments of systemic resistance of vascular and also systemic and pulmonary blood pressure are occurred ( 12 ). Recent studies have established DDAH2 has the main contribution to NO activity in endothelial cells. Consequently, *DDAH2* gene silencing decreases about 40% endothelial relaxation ( 13 ). Confirming recent studies the prevalence of the dysfunctional *DDAH2* gene variants could make enzyme activity variants that affect the risk of MetS related diseases. Seo and cols. reported that SNP rs2272592 of *DDAH2* in contrast to SNP rs805304 is in the relation to type 2 diabetes ( 11 ), while the rs805304 C allele was connected to the risk of myocardial infarction and obesity decrement ( 14 ). As reported by Xuan and cols., patients with coronary artery disease exhibited a significant correlation of the *DDAH2* genetic polymorphism (-499C/G, rs805305) with plasma ADMA ( 15 ).

Taking into account the crucial role of hypertension and diabetes in MetS, as well as inconclusive results about the association between MetS and *DDAH2* , it appeared that genetic polymorphism in *DDAH2* may contain biomarkers for the correlation of MetS. Therefore, this study's purpose was mainly to assess the association of the DDAH2 polymorphism with the risk of MetS among the Azar cohort population.

## SUBJECTS AND METHODS

### Subjects

In this case-control study, 626 adult subjects, including 332 with MetS and 294 healthy controls drawn from the Azar Cohort study, the large Iranian prospective epidemiological research study (Persian Cohort), were included ( 16 ). More details of this study were reported previously ( 17 ). Participants with at least three items of MetS criteria as cases or healthy volunteers as controls and aged 35-50 years old were included. Individuals who smoked or used hookah, drug abuse (addiction), and drank alcohol were excluded. Participants have been notified of the purpose of the study and then written their consent. The Ethics Committee of the Tabriz University of Medical Sciences (confirmation code: IR.TBZMED.REC.1399.257) was confirmed the present research. Demographic characteristics were also collected using a questionnaire.

### Anthropometric factors, MetS components, and blood sampling

Using NIH guidelines, anthropometric factors, including weight, heights, and waist circumference, were measured, and using the formula kg/m^2^ the body mass index (BMI) was determined. Blood pressure (BP) was measured twice a day with 2-minute intervals in each arm in a sitting position after a 10-minute rest period using a mercury sphygmomanometer (Rudolf Richter; DE-72417; Germany). The averages of these two measurements were used as the daily systolic and diastolic blood pressure measurements. Following 12 hours of overnight fasting blood samples were collected. The enzymatic methods were used for measuring serum levels of fasting blood sugar (FBS), triglyceride (TG), total cholesterol (TC), and high-density lipoprotein (HDL) ( 16 ). Friedewald's formula was used for calculating Low-density lipoprotein (LDL) ( 18 ). Besides, 100 µL of the blood sample was frizzed at −80 °C for DNA extraction by using a blood DNA extraction kit (DNA Biotech, Iran).

### Metabolic syndrome definition

This study used the National Cholesterol Education Program Adult Treatment Panel III report criteria (ATP III) for selecting MetS participants ( 19 ). Participants with at least three of the following criteria were defined as MetS cases: waist circumference ≥ 102 cm for men and ≥ 88 cm for women, TG ≥ 150 mg/dL or drug treatment for elevated triglycerides as an alternate indicator, and HDL-C values of < 40 mg/dL for men and < 50 mg/dL for women. Hypertension was defined as systolic blood pressure ≥ 130 mmHg and/or diastolic ≥ 85 mmHg or the use of antihypertensive medication. Elevated fasting glucose was considered to be ≥ 100 mg/dL or the use of glucose-lowering medication.

### PCR-RFLP and SNP genotyping

The genotyping of SNP rs805305 was performed by the PCR-RFLP method using specific forward, 5′-CCTTCTCGTTCGGGTATTCAG-3′; and reverse, 5′-TCCAGACCTTCCGCTCCT-3′ primers and restriction enzyme SmaI. Briefly, to amplify the fragments, we used 20 µL of PCR reaction mix including 10 µL Master Mix RED (5200300-1250: Ampliqon, Denmark), 0.5 µL (10 pmol/µL) of each forward and reverse primers (Bioneer, Takapouzist, Iran), 2 µL of the extracted DNA (50 ngr/µL) as a template and 7.5 µL deionized water. Thermal cycling was performed in a Primus 96 advanced thermal cycler (PEQLAB, Erlangen, Germany) under the following conditions: an initial hot start at 94 °C for 5 min, followed by 35 cycles of denaturation at 94 °C for 60 sec, annealing at 58 °C for 45 sec, and extension at 72 °C for 40 sec, and final elongation at 72 °C for 5 min. The amplified PCR product (4 μL) was digested with 2 μL SmaI (BioLabs; UK) in 2 μL 10X buffer with deionized water to a final volume of 20 μL in a 30 °C water bath for 4 h. After digestion, the treated mixture was electrophoresed on 2% agarose gel, and alleles (G: 341 bp; C: 254 and 87 bp) were visualized by dual intensity transilluminator (UVP, Upland, USA). Quality control genotyping was done using blind test assessments. Moreover, to verify the reproducibility of the results a 5% random sample of participants was genotyped twice by different operators ( 20 ).

### Statistical analysis

Data are presented as mean ± SD for numeric variables and frequency (percentage) for qualitative ones. Between groups, an independent t-test was used for quantitative variables comparison and chi-square tests for qualitative one's comparison. The χ2 test was applied for assessing Deviation from the Hardy-Weinberg equilibrium. Logistic regression analyses were used by adjusting for age and sex and displayed as odds ratios (ORs) and 95% confidence intervals (CIs). A two-tailed P _value_ ≤ 0.05 was considered significant. Statistical analyses were performed by using SPSS software version 17 (Chicago, IL, USA).

## RESULTS

Baseline characteristics of participants were stratified by case group and control group, and these are displayed in Table 1 . Of 332 participants with MetS, 42.5% were men, and 57.5%were women. The control group (294 participants) included 40.8% men and 59.2% women. Table 1 displayed the significant differences between the case and control groups regarding anthropometric parameters and serum levels of MetS components and also between males and females ( *P* < 0.001). Additionally, significant differences were seen in the serum levels of LDL between groups ( *P* ≤ 0.01), ( Table 1 ).

**Table 1:** MetS components and anthropometric factors of the participants

Parameters	MetS Patients (n = 332)	Controls (n = 294)	Pvalue
Total	mean ± SD	mean ± SD	
Age (years)	43.05 ± 4.44	41.70 ± 4.50	<0.001
Male n(%)	141 (42.5)	120 (40.8)	
Female n(%)	191 (57.5)	174 (59.2)	
Weight (kg)	85.11 ± 13.37	74.07 ± 13.16	<0.001
BMI (kg/m^2^)	32.01 ± 4.18	27.83 ± 4.93	<0.001
Waist circumference (cm)	99.33 ± 9.22	87.93 ± 10.19	<0.001
SBP (mmHg)	119.45 ± 14.49	111.95 ± 12.83	<0.001
DBP (mmHg)	79.22 ± 8.89	73.11 ± 8.59	<0.001
Glucose (mg/dL)	100.03 ± 14.97	91.35 ± 9.49	<0.001
Total cholesterol (mg/dL)	204.92 ± 43.53	187.48 ± 33.72	<0.001
LDL (mg/dL)	121.04 ± 34.45	114.59 ± 32.13	0.01
HDL (mg/dL)	40.27 ± 9.73	49.68 ± 11.59	<0.001
Triglycerides (mg/dL)	215.3 ± 120.88	120.23 ± 41.57	<0.001
**Male**	**N = 141**	**N = 120**	
Age (years)	42.60 ± 4.40	42.29 ± 4.45	0.65
Weight (kg)	91.05 ± 13.79	77.21 ± 11.44	<0.001
BMI (kg/m^2^)	31.00 ± 3.84	25.96 ± 3.54	<0.001
Waist circumference (cm)	100.21 ± 9.22	88.10 ± 9.89	<0.001
SBP (mmHg)	121.98 ± 14.13	115.77 ± 14.09	<0.001
DBP (mmHg)	82.05 ± 9.88	75.57 ± 8.99	<0.001
Glucose (mg/dL)	103.02 ± 18.74	96.01 ± 8.96	<0.001
Total cholesterol (mg/dL)	205.54 ± 47.39	191.75 ± 37.58	0.01
LDL (mg/dL)	120.10 ± 42.86	119.16 ± 34.50	0.84
HDL (mg/dL)	36.09 ± 7.82	46.61 ± 10.86	<0.001
Triglycerides (mg/dL)	250.20 ± 153.94	129.83 ± 55.23	<0.001
**Female**	**N = 191**	**N = 174**	
Age (years)	43.26 ± 4.46	41.43 ± 4.52	<0.001
Weight (kg)	80.75 ± 11.30	71.90 ± 13.85	<0.001
BMI (kg/m^2^)	32.72 ± 4.30	29.11 ± 5.34	<0.001
Waist circumference (cm)	97.99 ± 9.01	87.90 ± 10.56	<0.001
SBP (mmHg)	117.61 ± 14.50	109.31 ± 11.18	<0.001
DBP (mmHg)	77.17 ± 7.44	71.42 ± 7.90	<0.001
Glucose (mg/dL)	97.79 ± 11.04	88.14 ± 8.48	<0.001
Total cholesterol (mg/dL)	204.67 ± 40.56	185.97 ± 34.06	<0.001
LDL (mg/dL)	123.27 ± 33.69	111.44 ± 30.09	0.001
HDL (mg/dL)	43.06 ± 7.82	51.79 ± 11.64	<0.001
Triglycerides (mg/dL)	191.60 ± 82.05	113.62 ± 26.86	<0.001

BMI: body mass index; WC: waist circumference; SBP: systolic blood pressure; DBP: diastolic blood pressure; HDL: high-density lipoprotein; LDL: low-density lipoprotein.

*P_value_ < 0.05.

Allele distribution and genotype frequency and association of *DDAH2* with MetS are shown in Table 2 . Quality control test by repeating the genotyping for 5% randomly selected DNA samples did not disagree with the outcome of original genotyping. The higher distribution of G allele and CG genotype in the control groups showed a significant association between this genotype and a lower risk of MetS even after adjusting for age and gender ( *P* ≤ 0.001). The genotypes frequencies of the total, male, and female samples in the control group had non-significant deviation ( *P* > 0.05) from Hardy-Weinberg Equilibrium (HWE) ( Table 3 ). Moreover, the G dominant genetic model (GG+GC) as an SNP rs805305 subtype, showed a significantly reduced risk of MetS ( *P* = 0.001) ( Table 2 ). Similarly, an increased percentage of CG genotype had a significantly lower risk of MetS in both males and females ( Table 2 ). Although the G dominant genotype (GG+GC) significantly reduced the MetS risk in males, before adjusting for age, and in females after adjusting for age, the C dominant model (GC+CC) showed non-significant effects.

**Table 2:** Association of DDAH2 c. −449 C/G SNP with MetS

Genotype/Allele	MetS Patients N (%)	Controls N (%)	Unadjusted Odds Ratio (95% CI)	Pvalue	* Adjusted Odds Ratio (95% CI)	Pvalue
**Total (N = 626)**	(N = 332)	(N = 294)				
CC	130 (39.2)	80 (27.4)		Reference		
CG	133 (40.1)	158 (54.1)	0.51 (0.36-0.74)	<0.0001	0.50 (0.34-0.74)	0.001
GG	69 (20.8)	54 (18.5)	0.78 (0.5-1.23)	0.29	0.76 (0.45-1.29)	0.32
C	393 (59.2)	313 (53.6)		Reference		
G	271 (40.8)	271 (46.4)	0.74 (0.63-0.99)	0.04	0.67 (0.54-0.86)	0.002
**(GG+GC) vs. CC**	202 (60.8) vs. 130 (39.2)	212 (72.6) vs. 80 (27.4)	0.58 (0.41-0.82)	0.002	0.56 (0.39-0.81)	0.002
HWE	X^2^ = 9.69; P < 0.005	X^2^ = 2.41; P > 0.05				
**Male (n = 259)**	(N = 141)	(N = 118)				
CC	45 (31.9)	23 (19.5)		Ref.		
CG	61 (43.3)	68 (57.6)	0.45 (0.24-0.84)	0.01	0.49 (0.24-0.98)	0.04
GG	35 (24.8)	27 (22.9)	0.66 (0.32-1.34)	0.25	0.73 (0.28-1.87)	0.52
C	151 (53.5)	109 (46.4)		Ref.		
**G**	131 (46.5)	127 (53.8)	0.74 (0.52-1.05)	0.09	0.89 (0.60-1.33)	0.58
(GG+GC) vs. CC	96 (68.1) vs. 45 (31.9)	95 (80.5) vs. 23 (19.5)	0.51 (0.29-0.92)	0.02	0.54 (0.28-1.04)	0.06
HWE	X2 = 2.4; P > 0.05	X2 = 2.79; P > 0.05				
**Female (n = 365)**	(N = 191)	(N = 174)				
CC	85 (44.5)	57 (32.8)		Ref.		
**CG**	72 (37.7)	90 (51.7)	0.53 (0.34-0.84)	0.008	0.51 (0.32-0.82)	0.006
GG	34 (17.8)	27 (15.5)	0.84 (0.46-1.54)	0.58	0.78 (0.41-1.49)	0.46
C	242 (63.4)	204 (58.6)	Ref.			
**G**	140 (36.6)	144 (41.4)	0.82 (0.6-1.10)	0.19	0.55 (0.40-0.77)	<0.0001
(GG+GC) vs. CC	106 (55.5) vs. 85 (44.5)	117 (67.2) vs. 57 (32.28)	0.60 (0.39-0.93)	0.22	0.57 (0.36-0.89)	0.01
HWE	X2 = 6.76; P < 0.01	X2 = 0.76; P > 0.05				

* Adjusted odds ratio (95% CI) for age and sex in total sample of study; adjusted for age in both gender. *P < 0.05.

**Table 3:** HWE estimation for control groups

	Total		Male		Female	
	*Observed #	Expected #	*Observed #	Expected #	*Observed #	Expected #
CC	80	86.6	23	27.5	57	59.8
CG	158	144.8	68	58.9	90	84.4
GG	54	60.6	27	31.5	27	29.8
Chi-squared value	2.410		2.794		0.762	
P value	0.121		0.095		0.383	

## DISCUSSION

Further investigation is required around the effects of variant genotypes on the risk of metabolic syndrome as a public health problem and related diseases. In this case, the apparent mechanism of the polymorphism function of *DDAH2* in MetS is mainly unclear. Therefore, the current study evaluated the role of −499C/G, rs805305 polymorphism in the *DDAH2* gene on MetS. As far as our knowledge is concerned, this is the first study that examines the relationship between 499C/G, rs805305 polymorphism of the *DDAH2* gene, and the risk of MetS. As would be expected, the present study demonstrated substantial differences in MetS components between MetS cases and controls with and without gender classification. Moreover, the finding indicated that carriers of G allele, GC genotype, and G dominant genetic model, (GG+GC) compared to CC, manifested a lower risk of MetS compared to individuals conveying genotype GG, and C recessive genetic model GG vs (GC+CC). The main concern regarding the finding of this study is the fact that the G allele is significantly associated with the decreased odds ratios of MetS in total and female samples. Also, in the male subpopulation, the odds ratio of the G allele was lower than 1 but insignificant. However, although the odds ratios of GG genotype in total, male, and female samples were decreasing (0.76, 0.73, and 0.78 respectively) their associations were non-significant. It is probably was due to sample sizes. Pérez-Hernández and cols. found that the rs805304 C allele of *DDAH2* plays a protective role in patients with myocardial infarction. Moreover, they opined that this allele of the rs805304 polymorphism was related to the risk of obesity reduction as well ( 14 ). On the other hand, a non-significant association was shown between the CC genotype of SNP rs805304 (-1151 C/A) and its dominant genotype model (AG+GG) and diabetes and hypertension ( 11 ). Maas and cols. declared that the polymorphisms of −1151 A/C and −449 G/C located on the *DDAH2* promoter region were associated with the prevalence of hypertension enhancement ( 21 ). It is acknowledged that the G allele of the *DDAH2* gene − 499 C/G polymorphism is a major risk factor for male Egyptian CAD patients with 35-50-year-old ( 22 ). Although a significant relation was exhibited between the higher plasma level of ADMA and − 499 C/G rs 805305 polymorphism, no relation was reported between the *DDAH* 2 polymorphisms and the risk of CAD ( 15 , 23 ). This inconsistency may be ascribed to sample size insufficiency, or different susceptibility, ethnic diversity, or environmental factors, and their impact on genes. Evidence suggests that DDAH2 via some mechanisms has revealed a protective role on MetS.

Previous studies indicated the common effects of DDAH2 on hypertension, diabetes, CVD is attributed to ADMA. It has been reported that high levels of ADMA are associated with hypertension, type2 diabetes, and insulin resistance ( 24 , 25 ). DDAH2 is the main regulator of ADMA levels, a decreased level of *DDAH2* expression that can lead to an increase in ADMA concentration ( 15 ). High serum ADMA levels through various mechanisms resulted in an increased risk of metabolic syndrome components. In this way, Chen and cols. alleged that dose-dependent incubation of oxidized low-density lipoprotein (oxLDL) decreased DDAH2 protein expression. In contrast, the increment level of ADMA was induced by oxLDL. They concluded that the DDAH2/ADMA system during the transformation of macrophage foam cells may regulate lipid metabolism; additionally, its protective role may observe in deregulated lipid metabolism of foam cells of macrophages ( 26 ).

The strength of the current study is a sample size selected from the cohort population of the same ethnicity. Moreover, this is the first study that examined the relationship between 499C/G, rs805305 polymorphism in the *DDAH2* gene, and MetS. Also, we recently reported the risk-increasing role of *NOS3* -c.894G>T in MetS in the Azar-cohort population, which affects another component of the NO pathway, the eNOS enzyme ( 27 ). Despite these strong points, this study has some limitations. First, the cross-sectional design of the research was allowing no causal interferences. Second, the relation of other polymorphisms of MetS was not examined. Besides, for the reason the ADMA levels and nitric oxide activity were not measured, reporting the mechanisms that underlying the relationship between *DDAH2* gene variation and MetS should be inferred with caution. Finally, the small sample size was another limitation of this study. The effect of this limitation was seen especially when we found that although there was a significant association with the G allele, the GG genotype was not associated significantly ( Table 2 ). However, by testing analysis for a hypothetical case and control groups with doubled size (Controls: CC: 160, CG: 300, GG: 316; Cases: CC: 260, CG: 266, GG: 138) we can see that the ORs are lower than 1 for all comparisons as we reported, however, the GG genotype also shows the significant effect ( *P* < 0.0001) on decreased OR (data not shown).

In conclusion, according to these study findings, the G allele and CG genotype of *DDAH2* rs805305 polymorphism and its G dominant genotype model is significantly associated with a lower risk of MetS. Considering gene-gene interactions, further research is needed to elucidate the implication of other gene variants of *DDAH2* on MetS criteria to provide more accurate results in diverse ethnic populations. Considering that SNPs association studies are prone to spurious associations by chance, therefore it is suggested that larger samples size could be evaluated and replication in other groups could be tried.

## References

[1] Aslan Çin NN, Yardımcı H, Koç N, Uçaktürk SA, Akçil Ok (2020). M. Triglycerides/high-density lipoprotein cholesterol is a predictor similar to the triglyceride-glucose index for the diagnosis of metabolic syndrome using International Diabetes Federation criteria of insulin resistance in obese adolescents: A cross-sectional study. J Pediatr Endocrinol Metab.

[2] Sherling DH, Perumareddi P, Hennekens CH (2017). Metabolic syndrome. J Cardiovasc Pharmacol Ther.

[3] Ajlouni K, Khader Y, Alyousfi M, Al Nsour M, Batieha A, Jaddou H (2020). Metabolic syndrome amongst adults in Jordan: prevalence, trend, and its association with socio-demographic characteristics. Diabetol Metab Syndr.

[4] Gündogan K, Bayram F, Capak M, Tanriverdi F, Karaman A, Ozturk A (2009). Prevalence of metabolic syndrome in the Mediterranean region of Turkey: evaluation of hypertension, diabetes mellitus, obesity, and dyslipidemia. Metab Syndr Relat Disord.

[5] Kalan Farmanfarma K, Kaykhaei MA, Adineh HA, Mohammadi M, Dabiri S, Ansari-Moghaddam A (2019). Prevalence of metabolic syndrome in Iran: A meta-analysis of 69 studies. Diabetes Metab Syndr.

[6] Gallagher EJ, Leroith D, Karnieli E (2011). The metabolic syndrome-from insulin resistance to obesity and diabetes. Med Clin North Am.

[7] DeBoer MD (2019). Assessing and managing the metabolic syndrome in children and adolescents. Nutrients.

[8] Pasaoglu OT, Bircan FS, Topal T, Turkozkan N (2021). Positive effects of melatonin on renal nitric oxide-asymmetric dimethylarginine metabolism in fructose-fed rats. Metab Syndr Relat Disord.

[9] Kaur K, Singh N, Dhawan RK (2019). Exploring the role of dimethylarginine dimethylaminohydrolase-mediated reduction in tissue asymmetrical dimethylarginine levels in cardio-protective mechanism of ischaemic postconditioning in rats. Iran J Basic Med Sci.

[10] Tran CT, Fox MF, Vallance P, Leiper JM (2000). Chromosomal localization, gene structure, and expression pattern of DDAH1: Comparison with DDAH2 and implications for evolutionary origins. Genomics.

[11] Seo HA, Kim SW, Jeon EJ, Jeong JY, Moon SS, Lee WK (2012). Association of the DDAH2 gene polymorphism with type 2 diabetes and hypertension. Diabetes Res Clin Pract.

[12] Teerlink T, Luo Z, Palm F, Wilcox CS (2009). Cellular ADMA: Regulation and action. Pharmacol Res.

[13] Pope AJ, Karuppiah K, Cardounel AJ (2009). Role of the PRMT-DDAH-ADMA axis in the regulation of endothelial nitric oxide production. Pharmacol Res.

[14] Pérez-Hernández N, Vargas-Alarcón G, Arellano-Zapoteco R, Martínez-Rodríguez N, Fragoso JM, Aptilon-Duque G (2014). Protective role of DDAH2 (rs805304) gene polymorphism in patients with myocardial infarction. Exp Mol Pathol.

[15] Xuan C, Xu LQ, Tian QW, Li H, Wang Q, He GW (2016). Dimethylarginine dimethylaminohydrolase 2 (DDAH 2) gene polymorphism, asymmetric dimethylarginine (ADMA) concentrations, and risk of coronary artery disease: A case-control study. Sci Rep.

[16] Poustchi H, Eghtesad S, Kamangar F, Etemadi A, Keshtkar AA, Hekmatdoost A (2018). Prospective epidemiological research studies in Iran (the Persian cohort study): Rationale, objectives, and design. Am J Epidemiol.

[17] Farhang S, Faramarzi E, Amini Sani N, Poustchi H, Ostadrahimi A, Alizadeh BZ (2019). Cohort profile: The AZAR cohort, a health-oriented research model in areas of major environmental change in Central Asia. Int J Epidemiol.

[18] Poustchi H, Eghtesad S, Kamangar F, Etemadi A, Keshtkar AA, Hekmatdoost A (2017). Prospective epidemiological research studies in Iran (the PERSIAN cohort study): Rationale, objectives, and design. Am J Epidemiol.

[19] Rezaianzadeh A, Namayandeh SM, Sadr SM (2012). National Cholesterol Education Program Adult Treatment Panel III Versus International Diabetic Federation Definition of Metabolic Syndrome, Which One is Associated with Diabetes Mellitus and Coronary Artery Disease?. Int J Prev Med.

[20] Green MR, Sambrook J (2012). Molecular cloning: A laboratory manual.

[21] Maas R, Erdmann J, Luneburg N, Stritzke J, Schwedhelm E, Meisinger C (2009). Polymorphisms in the promoter region of the dimethylarginine dimethylaminohydrolase 2 gene are associated with prevalence of hypertension. Pharmacol Res.

[22] Gad MZ, Hassanein SI, Abdel-Maksoud SM, Shaban GM, Abou-Aisha K (2011). Association of ddah2 gene polymorphism with cardiovascular disease in Egyptian patients. J Genet.

[23] Xu AG, Xu RM, Lu CQ, Li DD, Xu QF, Guo J (2012). Association study of dimethylarginine dimethylaminohydrolase 2 gene polymorphisms and coronary heart disease. Mol Med Rep.

[24] Qiu N, Wei XM, Zhang ZJ, He YL, Zhou XK, Xiong Y (2020). Asymmetrical dimethylarginine induces dysfunction of insulin signal transduction via endoplasmic reticulum stress in the liver of diabetic rats. Life Sci.

[25] Lee Y, Mehrotra P, Basile D, Ullah M, Singh A, Skill N (2021). Specific lowering of asymmetric dimethylarginine by pharmacological dimethylarginine dimethylaminohydrolase improves endothelial function, reduces blood pressure and ischemia-reperfusion injury. J Pharmacol Exp Ther.

[26] Chen CH, Zhao JF, Hsu CP, Kou YR, Lu TM, Lee TS (2019). The detrimental effect of asymmetric dimethylarginine on cholesterol efflux of macrophage foam cells: Role of the NOX/ROS signaling. Free Radic Biol Med.

[27] Seyedrezazadeh E, Faramarzi E, Bakhtiyari N, Ansarin A, Gilani N, Amiri-Sadeghan A (2021). Association of NOS3-c. 894G> T transversion with susceptibility to metabolic syndrome in Azar-cohort population: A case-control study and in silico analysis of the SNP molecular effects. Iran J Basic Med Sci.

